# Focal brain ischemia in mice does not cause electrophysiological signs of critical illness neuropathy

**DOI:** 10.1186/s13104-020-05248-2

**Published:** 2020-09-10

**Authors:** Petra Huehnchen, Klaus Viktor Toyka, Karen Gertz, Matthias Endres, Wolfgang Boehmerle

**Affiliations:** 1 Charité – Universitätsmedizin Berlin, Corporate Member of Freie Universität Berlin, Humboldt-Universität zu Berlin and Berlin Institute of Health, Klinik und Hochschulambulanz für Neurologie, 10117 Berlin, Germany; 2 Charité – Universitätsmedizin Berlin, Corporate Member of Freie Universität Berlin, Humboldt-Universität zu Berlin and Berlin Institute of Health, Cluster of Excellence NeuroCure, 10117 Berlin, Germany; 3 Berlin Institute of Health, Anna-Louisa-Karsch Str. 2, 10178 Berlin, Germany; 4 Department of Neurology, University of Würzburg, 97080 Würzburg, Germany; 5 Charité – Universitätsmedizin Berlin, Corporate Member of Freie Universität Berlin, Humboldt-Universität zu Berlin, and Berlin Institute of Health, Center for Stroke Resarch Berlin, 10117 Berlin, Germany; 6 German Center for Neurodegenerative Diseases (DZNE), 10117 Berlin, Germany; 7 DZHK (German Center for Cardiovascular Research), Partner Site Berlin, 10117 Berlin, Germany

**Keywords:** Stroke, Axonal degeneration, Electromyography, Critical illness, Mice

## Abstract

**Objective:**

Critical illness polyneuropathy (CIP) is a common complication of severe systemic illness treated in intensive care medicine. Ischemic stroke leads to an acute critical injury of the brain with hemiparesis, immunosuppression and subsequent infections, all of which require extended medical treatment. Stroke-induced sarcopenia further contributes to poor rehabilitation and is characterized by muscle wasting and denervation in the paralytic, but also the unaffected limbs. Therefore, we asked whether stroke leads to an additional CIP-like neurodegeneration.

**Results:**

Focal brain ischemia was induced in adult mice by 60-min middle cerebral artery occlusion (MCAo) following reperfusion and led to functional deficits and marked hemispheric brain atrophy. Nerve conduction function and muscle potentials were measured in the ipsilateral sciatic nerve and gastrocnemius and quadriceps muscle with electroneurography/-myography on days 10, 22, 44 after stroke. An additional crush-injury to the sciatic nerve was included in two sham mice as positive control (sham +). We found no differences in nerve conduction function nor spontaneous electromyographic activity between MCAo and sham animals. Sham + mice developed marked reduction of the motor action potential amplitudes and conduction velocities with pathologic spontaneous activity. In conclusion, we found no peripheral nerve dysfunction/degeneration as signs of a CIP-like phenotype after MCAo.

## Introduction

Critical illness polyneuropathy (CIP) is a frequent and severe neurological complication of intensive care treatment with up to one-third of critically ill patients being affected in clinical assessment (reviewed by [1]). CIP is characterized electrophysiologically by a decrease in the amplitudes of the compound motor nerve action potentials (CMAP) and sensory nerve action potentials (SNAP) while motor and sensory nerve conduction velocities (MCV, SCV) are only mildly altered [2]. Electromyography (EMG) shows fibrillations and positive sharp waves (PSW) as indicators of axonal degeneration. The underlying pathomechanisms of CIP remain largely elusive, but are multifactorial including abnormal microcirculation, hyperglycemia, mitochondrial dysfunction and inactivation of sodium channels. A catabolic state with muscle wasting, oxidative stress and protease activation were also observed [3, 4]. Artificial ventilation, multi-organ failure, deranged systemic inflammatory responses (SIRS/sepsis) are identified clinical risk factors [5]. Unfortunately, many such factors cannot be mimicked in preclinical models due to the complex nature of an intensive care treatment. High mortality rates of sepsis models [6] hinder investigation of long-term established hallmark parameters of neurodegeneration in the peripheral nervous system. Ischemic stroke is a frequent and acute critical injury to the brain often resulting in immobilization, inflammation and immunosuppression (reviewed by [7]). Stroke patients are prone to secondary infections, adding to the risk of a global critical illness [8] often in need for intensive care treatment [9]. Additionally, stroke-induced sarcopenia results in muscle wasting, denervation, remodeling, and atrophy, which were observed in the paralytic muscle of mice and stroke patients (reviewed by [10]). Interestingly, muscle weakness also occurs in the *unaffected* limb in hemiplegic stroke patients [11]. Furthermore, catabolic signaling and proteasome activity as well as markers for inflammation were found in the paralytic as well as the unaffected leg in murine stroke models [12, 13]. Therefore, we asked whether middle cerebral artery occlusion (MCAo) induces a CIP-like neurodegeneration, which could partially account for stroke-induced sarcopenia, and whether preclinical stroke models can serve as an alternative model system to study CIP.

## Main text

### Methods

For a more detailed description on the methods please refer to the Additional file 1.

### Functional assessment

A total of 30 male 20-week old C57Bl/6 mice (Charles River, Sulzfeld, Germany) were used. All experimental procedures followed local and national guidelines and were approved by a governmental agency (Landesamt für Gesundheit und Soziales Berlin). The general wellbeing of the mice and their functional deficits were assessed daily and rated according to the Bederson score adapted to mice: 0 = no deficits, 1 = flexion of forelimb, 2 = deviation from midline with circling in some animals, 3 = loss of postural reflexes [14].

### Methods against bias

Due to expected higher drop-out rates in the MCAo group [15], mice were allocated to the MCAo and sham group asymmetrically (MCAo: n = 23, sham: n = 7) using an online randomization tool (GraphPad Software, La Jolla, CA, USA). Two randomly selected sham mice received an additional 5 s crush injury of the ipsilateral sciatic nerve to induce acute nerve degeneration as internal positive control (sham +). The investigators conducting the experiments were blinded throughout the entire experimental period including analysis.

### Middle cerebral artery occlusion model (MCAo)

All surgery was performed by a previously trained experimenter according to a standard operating procedure [16] using the transient proximal MCAo filament model as previously described [17]. Reperfusion was induced by withdrawing the filament after an occlusion time of 60 min.

### Crush injury of the sciatic nerve

In anesthesia, the sciatic nerve was exposed at mid-thigh level and crushed with a constant pressure for 5 s using a non-serrated clamp. Afterwards, the clamp was released and the incision closed.

### Nerve conduction studies

CMAP and MCV of the sciatic nerve were measured with a customized Neurosoft Evidence 3102evo EMG/ENG device (Schreiber & Tholen Medizintechnik GmbH, Stade, Germany) under 3% isoflurane anesthesia with 50% O_2_ at days 10, 22 and 44 after surgery as described [18].

### Electromyography

Pathologic spontaneous activity (PSA) was assessed under 3% isoflurane anesthesia with 50% O_2_ by longitudinally inserting steel needle electrodes subcutaneously along the fascia over the belly of the ipsilateral gastrocnemius and quadriceps muscles. PSA consisting of (superimposed) positive sharp wave (PSW) and fibrillation potentials was recorded in each muscle 10 times over a 250 ms time period. Superimposed PSW were counted as “events” and quantified as previously described [18]. Spontaneous fasciculations and high frequency bursts were scored as follows: 0 = no fasciculations/high frequency bursts, 1 = few fasciculations/high frequency bursts, 2 = marked fasciculations/high frequency bursts. Spontaneous activity induced by extramuscular needle movements were scored according to duration: 0 = < 0.5 s, 1 = 0.5-1 s, 2 = > 2 s.

### Statistical analysis and data availability

Statistical analysis was performed using Prism v8.0 (GraphPad Software, La Jolla, CA). Gaussian distribution was checked prior to analysis with Shapiro–Wilk normality test. Normally distributed data were analyzed with unpaired two-sided t-tests (2 groups) or 2-way ANOVA with Holm-Sidak post hoc analysis (≥ 3 groups) and are presented as mean ± sem. Mann–Whitney-U (2 groups) or Kruskal–Wallis test with Dunn’s post hoc test (≥ 3 groups) were used for non-parametric data, which are displayed as violin plots. p < 0.05 was considered statistically significant. The analyzed dataset is available on Mendeley Data (Huehnchen & Boehmerle 2020, Mendeley Data, V1, 10.17632/9dkwv5w9b3.1, http://dx.doi.org/10.17632/9dkwv5w9b3.1) [19].

## Results

### Clinical course after 60-min MCAo/reperfusion in mice

**Fig. 1:**
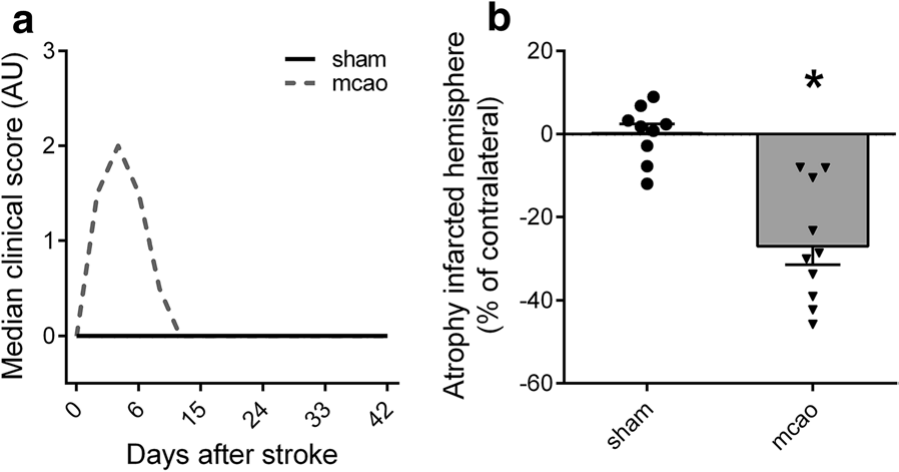
Clinical course after 60 min MCAo/reperfusion. **a** Brain ischemia induced clinical deficits corresponding to an increase of the median Bederson score values with a peak on day 3. None of the sham operated mice had an increased Bederson score; sham + mice with additional sciatic nerve crush injury showed a paralysed lower hind limb (not shown). **b** Histologic analysis of the brains revealed a marked atrophy of the infarcted hemisphere compared to the contralateral hemisphere. Statistical analyses: **a** Kruskal–Wallis test with Dunn’s method, in (**c**) unpaired two-sided t-test. * p < 0.05

We randomly assigned 23 male C57Bl/6 mice to 60 min MCAo/reperfusion and seven to the sham group. Focal brain ischemia was induced by occluding the middle cerebral artery with a monofilament for the duration of 60 min followed by reperfusion. Mice after MCAo/reperfusion showed distinct sensory-motor deficits with a median Bederson score of 2 at the peak of the brain edema around day 3 to 5 following surgery, but recovered quickly within 10 days after ischemic injury (Fig. 1a). Histologic analysis of brains from mice with MCAo/reperfusion on day 44 after stroke showed a marked reduction of brain volume (atrophy) of the ischemic hemisphere of − 27% ± 4% compared to the contralateral hemisphere (unpaired two-sided *t* test, p < 0.0001; Fig. 1b).

### MCAo/reperfusion does not lead to dysfunction of peripheral nerves

**Fig. 2:**
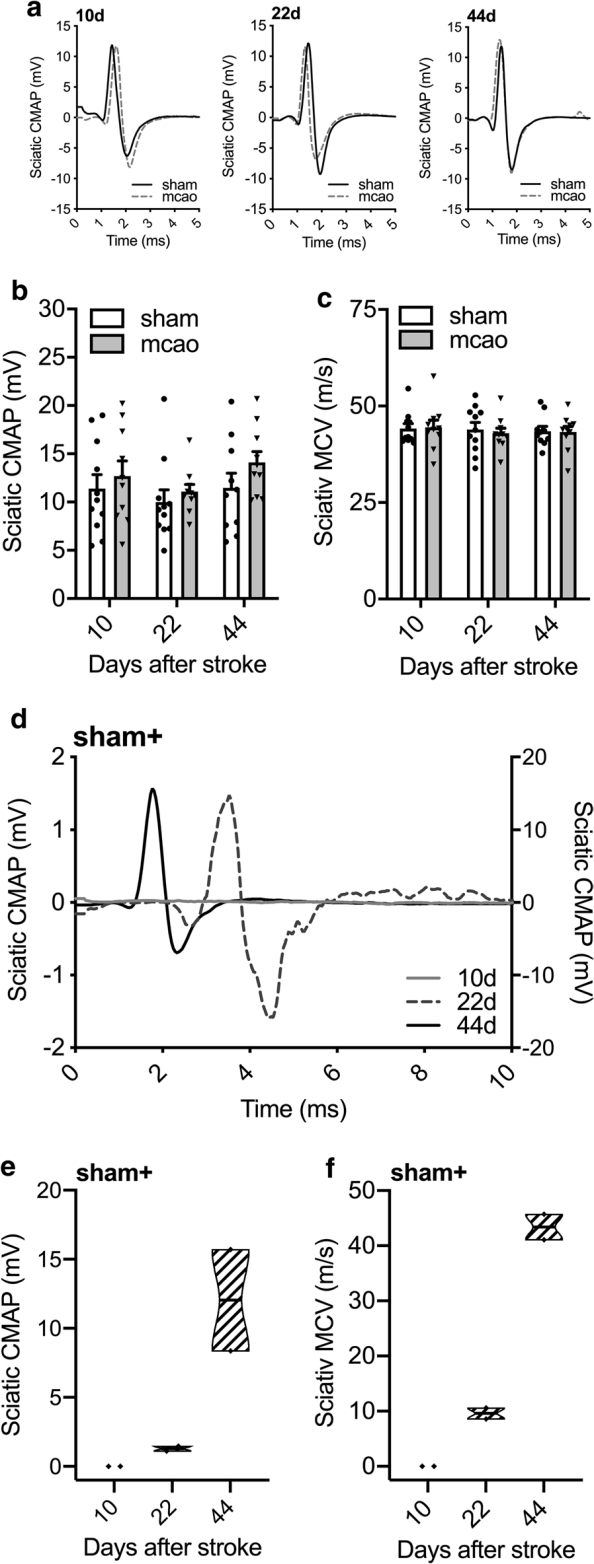
Serial nerve conduction velocity testing in the sciatic nerve after MCAo/reperfusion. **a** Representative recordings of CMAPs in the sciatic nerve obtained from mice after MCAo/reperfusion (grey dotted line) or sham operation (black solid line) on day 10 (left panel), 22 (middle panel), and 44 (right panel). **b** CMAP amplitude of MCAo and sham operated mice were comparable at all observed time points. **c** MCV remained unchanged in MCAo and sham-operated mice at all time points. **d** Representative sciatic nerve CMAP of a sham + mouse: CMAPs are virtually not detectable on day 10 (solid grey line, scale on left y-axis), severely decreased in its amplitude and delayed indicating slowed motor nerve conduction velocity with signs of temporal dispersion on day 22 (dashed grey line, scale on left y-axis) and fully regenerated on day 44 (solid black line, scale on right y-axis) after crush injury. **e** Crush injury to the sciatic nerve in sham + animals induced a severe decrease of the sciatic CMAP amplitude, which recovered by day 44. **f** The sciatic motor nerve conduction velocity (MCV) was severely decreased and steadily recovered in sham + mice. Statistical analysis: (**b**) 2-way ANOVA with Sidak post hoc, in (**c**) Kruskal–Wallis test with Dunn’s method

Mice were assessed for functional indicators of nerve dysfunction and degeneration of the ipsilateral sciatic nerve at days 10, 22 and 44 after stroke. We found no differences in CMAP amplitudes (2-way ANOVA, p = 0.70, Fig. 2a, b) nor in sciatic MCV (Kruskal–Wallis test, p > 0.99, Fig. 2c) between sham and MCAo operated mice.

Sham operated mice with an additional crush injury to the ipsilateral sciatic nerve (sham +) served as positive control demonstrating the electrophysiologic sequelae in an established type of experimentor-induced nerve degeneration. In these animals, we observed markedly smaller and delayed CMAPs of the sciatic nerve as early as at day 10, which gradually improved towards normal values via nerve fiber regeneration over time (Fig. 2d, e). The MCV of the injured sciatic nerve were decreased accordingly in sham + animals and likewise recovered over time (Fig. 2f).

### MCAo/reperfusion does not induce peripheral nerve degeneration in skeletal muscle

**Fig. 3:**
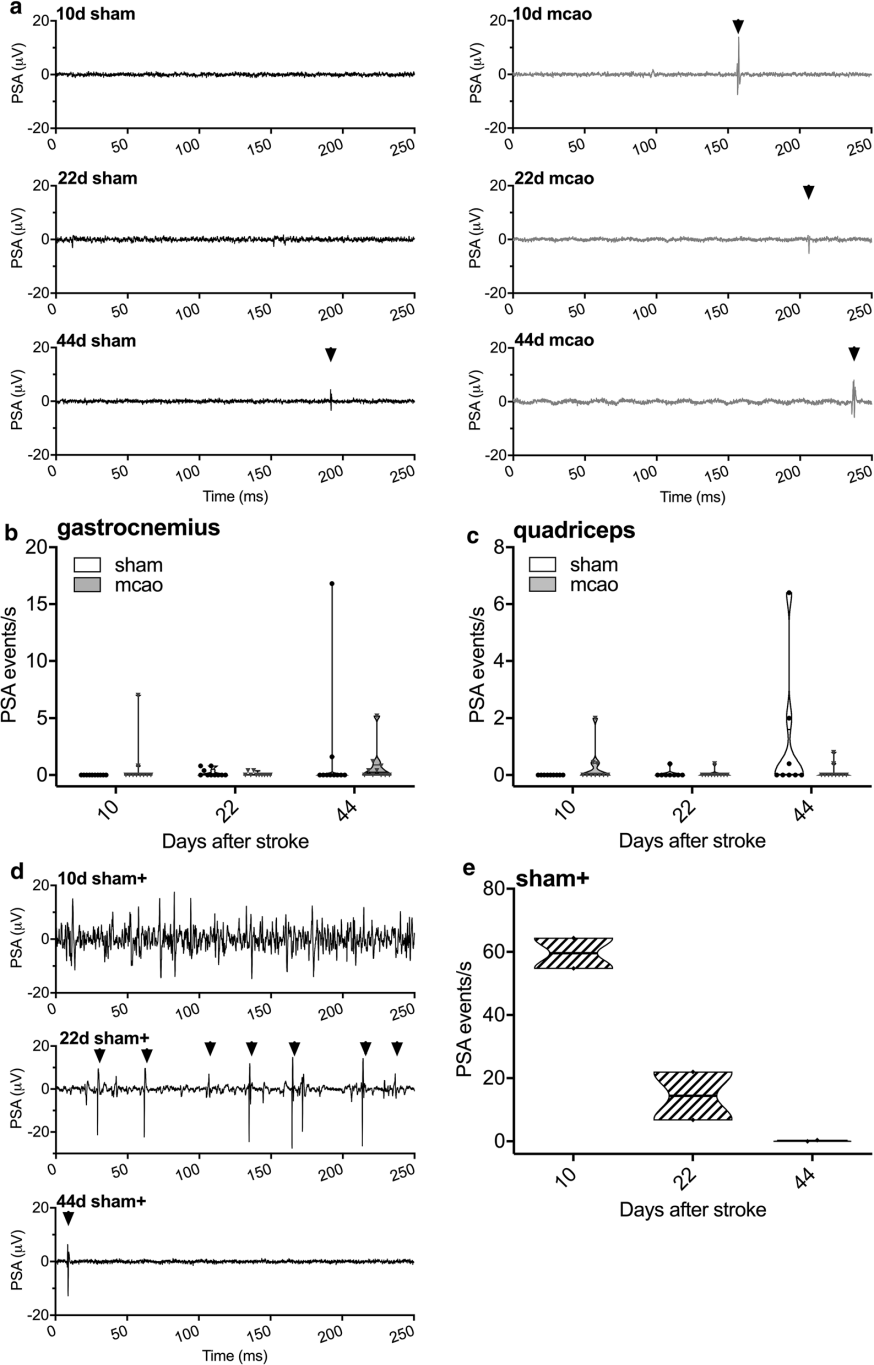
Serial electrophysiologic assessment of pathologic spontaneous activity in hind limb muscles after MCAo/reperfusion. **a** Representative sections from electromyographic recordings of the gastrocnemius muscle in mice with MCAo/reperfusion and sham operation on days 10, 22 and 44. Some isolated spontaneous potentials are marked by arrow heads in the traces recordings. **b** MCAo/reperfusion and sham-operated mice showed only rare fasciculation and fibrillation potentials and positive sharp waves at day 22 and more so at day 44 in the gastrocnemius muscle as well as the (**c**) quadriceps muscle. There was no significant difference between MCAo/reperfusion and sham operated mice in terms of PSA events. **d** Representative traces from electromyographic recordings of the gastrocnemius muscle in sham-operated mice with an additional crush injury to the sciatic nerve (sham + , positive control) on days 10, 22 and 44. At day 10, PSA was abundant and could only be quantified by lumping together superimposed fibrillation or fasciculation potentials and PSW as “events” of PSA. Note that PSW and fibrillation potentials become only discernable during partial recovery at day 22 (single spontaneous potentials marked by arrow heads). **e** Quantification of PSA after sciatic crush injury in sham + mice: PSA was abundant at day 10, much less so at day 22 and control values were reached at day 44. Statistical analysis: (**b**, **c**) Kruskal–Wallis test with Dunn’s method

Induction of MCAo/reperfusion did not lead to a denervation pattern in electromyographic muscle activity. An increase in PSA could not be found in the gastrocnemius muscle (Kruskal–Wallis test, p > 0.99, Fig. 3a, b) nor in the quadriceps muscles (Kruskal–Wallis test, p > 0.82, Fig. 3c) at any time, neither in MCAo mice nor in sham controls without additional crush lesion. In contrast, the crush injury of the sciatic nerve induced massive (superimposed) PSA (“events”) in the gastrocnemius muscle indicating profound peripheral nerve fiber degeneration. The number of PSA events in sham + mice was highest on day 10 and steadily declined thereafter to control levels at day 44 (Fig. 3d, e). Expectedly, crush injury of the sciatic nerve did not result in PSA in the quadriceps muscle, as it is innervated by the femoral nerve (data not shown).

We additionally rated the occurrence of spontaneous fasciculation potentials, prolonged post-needle movement activity and high frequency bursts to evaluate muscle hyperexcitability. Overall, all three phenomena were mostly absent in MCAo and sham operated mice (Additional file 1: Figure S1). As the only abnormality we observed a mildly increased post-needle movement activity in the gastrocnemius muscle of the MCAo mice and this was seen only on day 22 (Kruskal–Wallis test, p = 0.0068; Additional file 1: Figure S1C) and in the quadriceps muscle of the sham operated mice on day 44 (Kruskal–Wallis test, p = 0.0002; Additional file 1: Figure S1D). Overall, the biological significance of these observations are questionable.

## Discussion

In this study, we demonstrate that experimentally induced ischemic stroke does not induce a systemic degenerative neuropathy in the MCAo mouse model over the 44-day post-stroke observational period. The profound functional deficits and the extended brain lesions adequately reflect the severity of the ischemic damage and are in line with previous studies [20, 21]. We decided against a measurement of regional cerebral blood flow (rCBF) as a parameter of MCAo severity because we were interested in the effects of brain ischemia vs. sham operation (i.e.”yes vs. no”) and not MCAo group differences following treatment, where subtly distinctions in rCBF are much more critical. Even despite large infarctions, the affected health status with reduced mobility and a catabolic state of the mice [12], some of the previously claimed risk factors of CIP (reviewed by [1]), we could not detect signs of peripheral nerve dysfunction.

The feasibility of our electrodiagnostic approach utilizing neurography and needle EMG was shown in multiple rodent disease models, including autoimmune inflammatory neuropathies, hereditary neuropathies and spinal muscular atrophy models [18, 22–24]. We included the well-characterized crush injury model of acute denervation (sham +) as a positive control. Here, we observed the full pattern of a degenerating neuropathy. The timing of the electrophysiological testing was based on reported studies using this experimental approach [25, 26] and observations in human CIP patients [27, 28]. Therefore, our experimental protocol was suited for the study and we preclude that methodical errors could account for the negative finding.

## Limitations

One principal limitation of this study is that the prolonged 60 min MCAo/reperfusion caused a clinical deterioration in some mice, which led to the necessity of their sacrifice according to humane endpoints thereby hindering the study of post-acute effects in these mice. One may speculate that these most afflicted mice could have become more susceptible as to developing CIP, but there is no evidence from our study substantiating this hypothesis. One alternative may be to use shorter occlusion times, which still produce reliable striatal infarcts with moderate functional deficits [17], but are more suitable to study post-stroke recovery [29]. Another limitation is that mice after MCAo, even with prolonged ischemic times of 60 min, recover fairly quickly from stroke-induced functional deficits, which diverges from the human situation. Most young mice show complete functional recovery by day 7–10, aged mice by day 10–15 after ischemic injury in the 60 min MCAo/reperfusion model [30]. Therefore, it can be discussed whether a *transient* MCAo is sufficient to induce prolonged states of critical illness. However, about 25% of stroke patients receive thrombolysis or thrombectomy therapy and even spontaneous reperfusion/nonocclusion can be seen in up to 50% of patients 4 days after symptom onset [31]. A transient occlusion model therefore adequately reflects the clinical situation. The problem with *permanent* MCAo in mice however is, that mortality rates are very high, not unlike sepsis models, which makes long-term effects difficult to investigate.

As infection and a deranged immune response to the former (SIRS, sepsis) play important roles in the development of CIP, one can argue that the subsequent immune response frequently observed after MCAo is not sufficient to induce the necessary pathophysiological key changes that lead to CIP. To preclude these aspects in the future, one possibility might be to choose a shorter occlusion time of 30 min and combine it with the induction of a hyper-immune response (SIRS) in MCAo mice, thereby increasing the clinically proven risk factors for CIP development. However, the downside of this approach is yet again an increased mortality rate, which hinders the study of long-term effects such as CIP.

## Supplementary information

**Additional file 1: Figure S1.** Supplementary material and methods and supplementary figure 1.

## Data Availability

The datasets analyzed for this study can be found on Mendeley Data (Huehnchen & Boehmerle 2020, Mendeley Data, V1, 10.17632/9dkwv5w9b3.1, http://dx.doi.org/10.17632/9dkwv5w9b3.1) [19].

## Abbreviations

CIP: Critical illness polyneuropathy
CMAP: Compound motor action potential
EMG: Electromyography
ENG: Electroneurography
MCAO: Middle cerebral artery occlusion
MCV: Motor conduction velocity
PSA: Pathologic spontaneous activity
PSW: Positive sharp wave potential
rCBF: Regional cerebral blood flow
SCV: Sensory conduction velocity
SIRS: Systemic inflammatory response syndrome
SNAP: Sensory nerve action potential
