# Supplementary material for: Focal brain ischemia in mice does not cause electrophysiological signs of critical illness neuropathy
Source: BMC Res Notes. 2020 Sep 10;13:425. doi: 10.1186/s13104-020-05248-2 (PMC7488231; doi:10.1186/s13104-020-05248-2)
Supplement: Supplementary file 1 — Additional file 1: Figure S1. Supplementary material and methods and supplementary figure 1. [file 13104_2020_5248_MOESM1_ESM.docx]

***Additional file***

# **Material and methods**

### Animal numbers and housing conditions

We used a total of 30 male inbred 20-week old C57Bl/6 mice (Charles River, Sulzfeld, Germany) for this study. All experimental procedures were planned according to institutional and national guidelines and approved by the governmental agency (Landesamt für Gesundheit und Soziales Berlin). Upon arrival, mice were randomly assigned to cages and housed in groups of five in an enriched environment and allowed food and tap water ad libitum. Animals were maintained on a 12:12 hour light/dark cycle (7 am – 7 pm). The general wellbeing of the mice and their functional deficits were assessed daily. The functional deficits were rated using the Bederson score adapted to mice corresponding to the following definitions: 0 = no deficits, 1= flexion of forelimb, 2 = deviation from midline with circling in some animals, 3 = loss of postural reflexes [1]. The score defines grade 1 as mild, grades 2 as moderate and 3 as severe functional deficits.

### Sample sizes and methods of randomization and blinding

We determined the group size of this exploratory trial using G*Power 3 statistical software [2]. Primary endpoint of the study was the evaluation of pathologic spontaneous activity (PSA) on electromyographic testing from the ipsilateral gastrocnemius and quadriceps muscle. We purposefully chose this parameter because assessment is simple, can be highly standardized and because PSA is a well-established hallmark sign of PNS degeneration and can be observed as early as 10 days after injury. Based on our prior experience and our published observations [3], we expected a standard deviation of 25 % and aimed at showing abnormal biological effects of at least 15 % more than seen in the sham control mice. Using multivariate analysis with a desired power of 0.9 and an alpha level of 0.05, the calculated sample size of the experiment was 20 mice. As it was previously reported that long-term survival after stroke in the 60 min MCAo/ reperfusion model is reduced [4; 5; 6] due to the large infarctions and stroke severity, we conservatively calculated with a total sample size of 30 animals to maintain statistical power in case mice had to be sacrificed according to predefined humane endpoints before the final analysis. Mice were allocated to the MCAo and sham group asymmetrically (MCAo: n=23, sham: n=7) using an online randomization tool (GraphPad Software, La Jolla, CA, USA). Two randomly picked sham operated mice additionally received a five-second crush injury of the ipsilateral sciatic nerve at mid-thigh level immediately after sham operation to induce acute nerve degeneration of the sciatic nerve and PSA in the gastrocnemius muscle, which served as internal positive control (sham+) for our primary endpoint. The investigators conducting the functional assessment and electrophysiological testing were blinded throughout the entire experimental period as to the type of surgery. Histologic analysis revealed that five mice showed no signs of cerebral infarcts or brain atrophy after ischemia. These mice were re-allocated to the sham group for statistical analysis in all endpoints.

### Middle cerebral artery occlusion model (MCAo)

All surgery was performed by a previously trained experimenter according to a published standard operating procedure [7]. Ischemic stroke was induced using the transient proximal MCAo filament model as previously described [8]. In brief, mice were anaesthetized with 1.5 % (vol/vol) isoflurane (30 % O_2_ and 65 % N_2_O) and placed on a heating pad (Harvard Apparatus, Holliston, MA, USA). Rectal temperature was continuously measured and body temperature maintained at 37.5°C. A 20 mm long silicon rubber-coated monofilament (Doccol Corp, Sharon, MA, USA) was inserted in the left internal carotid artery and advanced up to the anterior cerebral artery, thereby blocking blood flow to the middle cerebral artery. Filaments varied in diameter depending on bodyweight and size of the animal (0.06-0.09 mm in diameter for mice <30 g bodyweight, and 0.09-0.11 mm in diameter for mice >30 g bodyweight). Reperfusion was induced by withdrawing the filament after an occlusion time of 60 min. The 60 min MCAo was chosen as a well-characterized and standardized model of stroke, which regularly leads to striatal and cortical infarctions with contralateral hemiparesis and functional deficits. To detect the potential development of CIP, a longer than usual observational period including the acute and recovery phase after MCAo was chosen. Sham operations were done accordingly without filament insertion. Mice were placed in a heated cage during MCAo with a recovery period of 2 h following reperfusion.

### Crush injury of the sciatic nerve

Crush injury of the ipsilateral sciatic nerve was induced in two randomly selected mice of the sham group immediately after sham operation while still under anesthesia. A small incision was made at the hind mid-thigh level, the sciatic nerve exposed and crushed with a constant pressure for 5 s using a non-serrated clamp. Thereafter, the clamp was released, and the incision closed with sutures.

### Nerve conduction studies

Compound motor nerve action potentials (CMAP) and motor nerve conduction velocities (MCV) of the sciatic nerve were measured with a customized Neurosoft Evidence 3102evo EMG/ENG device (Schreiber & Tholen Medizintechnik GmbH, Stade, Germany) under 3 % isoflurane (vol/vol) anesthesia with 50 % O_2_ at days 10, 22 and 44 after surgery as previously described [3]. In brief, the tibial and peroneal nerves were stimulated with supramaximal currents at the ankle with steel electrodes (distal) and the total sciatic nerve was stimulated at the sciatic notch with modified Buchthal near-nerve electrodes (proximal, kindly provided by C. Krarup, Copenhagen, Denmark). CMAPs were recorded with steel needle electrodes in the foot muscles. MCV was calculated by measuring the distance between the distal and proximal stimulation electrodes.

### Electromyography

Pathologic spontaneous activity (PSA) was assessed under 3 % isoflurane (vol/vol) anesthesia with 50 % O_2_ with the same EMG machine by longitudinally inserting the 1 cm long steel needle electrodes subcutaneously 2 mm apart along the fascia over the belly of the ipsilateral gastrocnemius and quadriceps muscles. This electrode positioning avoids traumatic needle injuries within the muscle tissue. PSA consisting of positive sharp wave (PSW) and fibrillation potentials, but also superimposed multiple PSW or fibrillation potentials, was recorded in each muscle 10 times over a 250 ms time period (filter settings at 200 Hz and 5000 Hz). Single PSW and fibrillation potentials are easily recognized in mild axonal degeneration. With moderate to severe nerve degeneration, irregularly firing complex discharges of many superimposed PSW and fibrillation potentials cannot be differentiated into its individual components. Therefore these denervation-associated complex bursts were named “events” and quantified as events per second and calculated as previously described [3]. Utilizing this approximation method implies a bias towards underestimating the true number of PSW and fibrillation potentials. Reproducible identification and quantification of PSA events was verified offline from all completely stored recordings via a repeated analysis of the original data sets by an investigator (KVT) unaware of the group allocation. Additionally, spontaneous fasciculations and high frequency bursts were scored according to the following system: 0 = no fasciculations or high frequency bursts, 1 = few fasciculations or high frequency bursts, 2 = marked fasciculations or high frequency bursts. We also rated the spontaneous activity induced by extramuscular needle movements as follows: 0 = <0.5 s, 1= 0.5-1 s, 2 = >2 s.

### Statistical analysis and data availability

Normally distributed data are presented as mean ± sem with individual values depicted where appropriate. Non-parametric data is presented as violin plots. Data processing and all analyses were completed before unblinding of the examiners. Gaussian distribution of data was checked prior to statistical analysis using Shapiro-Wilk normality test. Statistical analysis was performed using Prism v8.0 (GraphPad Software, La Jolla, CA). Normally distributed data were analyzed using unpaired two-sided t-tests (2 groups) or 2-way ANOVA with Holm-Sidak post hoc analysis for multiple comparisons (3 groups over time). Not normally distributed data were analyzed with Mann-Whitney-U Test (2 groups) or Kruskal-Wallis test with Dunn’s post hoc test (3 groups). p<0.05 was considered statistically significant and is depicted by an asterisk (NS: not significant). The datasets analyzed for this study can be found on Mendeley Data (Huehnchen & Boehmerle 2020, Mendeley Data, V1, doi: 10.17632/9dkwv5w9b3.1, http://dx.doi.org/10.17632/9dkwv5w9b3.1) [9].

# **Supplementary Figures**


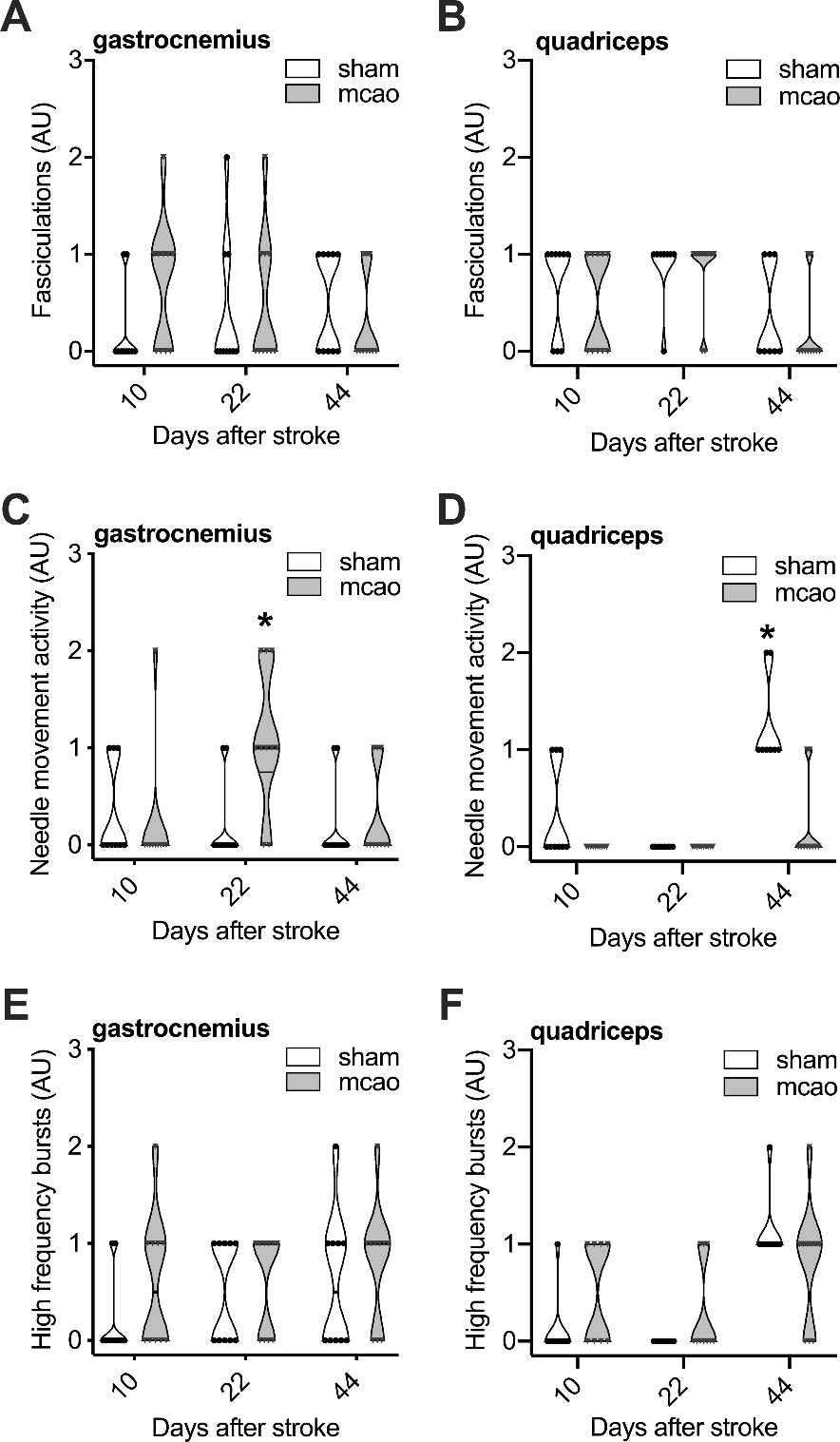


**Figure S1: Occurrence of fasciculations, post needle movement activity and high frequency bursts after severe ischemia.**

Fasciculations were rated by a scoring system from 0=absent (0) to 2=markedly prominent in the (A) gastrocnemius and (B) the quadriceps muscle. We did not detect any differences between sham and MCAO operated mice. Needle movement-induced activity was rated according to the duration of activity and was ranked from 0= <0.5 s to 2= >2 s. (C) In the gastrocnemius muscle, MCAo mice showed a prolonged post needle movement activity at day 22 when compared to sham-operated mice, but no differences were seen on day 44. (D) In the quadriceps muscle, we observed a prolonged post needle movement activity on day 44 in the sham group. High frequency bursts were also scored from 0=absent to 2=markedly prominent. We did not see any differences between sham and MCAo operated mice on any of the time points in the (E) gastrocnemius and (F) quadriceps muscle. Statistical analysis: (A-F) Kruskal-Wallis test with Dunn’s method. * p<0.05

# **References**

[1] J.B. Bederson, L.H. Pitts, M. Tsuji, M.C. Nishimura, R.L. Davis, and H. Bartkowski, Rat middle cerebral artery occlusion: evaluation of the model and development of a neurologic examination. Stroke 17 (1986) 472.

[2] F. Faul, E. Erdfelder, A.G. Lang, and A. Buchner, G*Power 3: a flexible statistical power analysis program for the social, behavioral, and biomedical sciences. Behav Res Methods 39 (2007) 175-91.

[3] F. Krieger, N. Elflein, S. Saenger, E. Wirthgen, K. Rak, S. Frantz, A. Hoeflich, K.V. Toyka, F. Metzger, and S. Jablonka, Polyethylene glycol-coupled IGF1 delays motor function defects in a mouse model of spinal muscular atrophy with respiratory distress type 1. Brain 137 (2014) 1374-93.

[4] D. Yuan, C. Liu, J. Wu, and B. Hu, Nest-building activity as a reproducible and long-term stroke deficit test in a mouse model of stroke. Brain Behav 8 (2018) e00993.

[5] G. Zhang, L. Chen, L. Yang, X. Hua, B. Zhou, Z. Miao, J. Li, H. Hu, M. Namaka, J. Kong, and X. Xu, Combined use of spatial restraint stress and middle cerebral artery occlusion is a novel model of post-stroke depression in mice. Scientific Reports 5 (2015) 16751.

[6] C. Meisel, K. Prass, J. Braun, I. Victorov, T. Wolf, D. Megow, E. Halle, H.D. Volk, U. Dirnagl, and A. Meisel, Preventive antibacterial treatment improves the general medical and neurological outcome in a mouse model of stroke. Stroke 35 (2004) 2-6.

[7] U. Dirnagl, and M.o.t.M.-S. Group, Standard operating procedures (SOP) in experimental stroke research: SOP for middle cerebral artery occlusion in the mouse. Nature Precedings (2012) 1–14.

[8] O. Engel, S. Kolodziej, U. Dirnagl, and V. Prinz, Modeling Stroke in Mice - Middle Cerebral Artery Occlusion with the Filament Model. Journal of Visualized Experiments : JoVE (2011) 2423.

[9] P. Huehnchen, and W. Boehmerle, Focal brain ischemia in mice does not cause electrophysiological signs of critical illness neuropathy. Mendeley Data V1 (2020): http://dx.doi.org/10.17632/9dkwv5w9b3.1.
